# Enzyme Nanosheet-Based Electrochemical Aspartate Biosensor for Fish Point-of-Care Applications

**DOI:** 10.3390/mi13091428

**Published:** 2022-08-29

**Authors:** Thenmozhi Rajarathinam, Dinakaran Thirumalai, Sivaguru Jayaraman, Seonghye Kim, Minho Kwon, Hyun-jong Paik, Suhkmann Kim, Mijeong Kang, Seung-Cheol Chang

**Affiliations:** 1 Department of Cogno-Mechatronics Engineering, College of Nanoscience and Nanotechnology, Pusan National University, Busan 46241, Korea; 2 Department of Chemistry, Pusan National University, Busan 46241, Korea; 3 Department of Polymer Science and Engineering, Pusan National University, Busan 46241, Korea

**Keywords:** amperometric biosensor, fish point of care, aspartate, enzyme nanosheets, olive flounder

## Abstract

Bacterial infections in marine fishes are linked to mass mortality issues; hence, rapid detection of an infection can contribute to achieving a faster diagnosis using point-of-care testing. There has been substantial interest in identifying diagnostic biomarkers that can be detected in major organs to predict bacterial infections. Aspartate was identified as an important biomarker for bacterial infection diagnosis in olive flounder (*Paralichthys olivaceus*) fish. To determine aspartate levels, an amperometric biosensor was designed based on bi-enzymes, namely, glutamate oxidase (GluOx) and aspartate transaminase (AST), which were physisorbed on copolymer reduced graphene oxide (P-rGO), referred to as enzyme nanosheets (GluOx-ASTENs). The GluOx-ASTENs were drop casted onto a Prussian blue electrodeposited screen-printed carbon electrode (PB/SPCE). The proposed biosensor was optimized by operating variables including the enzyme loading amount, coreactant (α-ketoglutarate) concentration, and pH. Under optimal conditions, the biosensor displayed the maximum current responses within 10 s at the low applied potential of −0.10 V vs. the internal Ag/AgCl reference. The biosensor exhibited a linear response from 1.0 to 2.0 mM of aspartate concentrations with a sensitivity of 0.8 µA mM^−1^ cm^−2^ and a lower detection limit of approximately 500 µM. Moreover, the biosensor possessed high reproducibility, good selectivity, and efficient storage stability.

## 1. Introduction

Point-of-care (POC) diagnostic devices are rapid, inexpensive, simple to use, and instrument independent. An idealized POC device can operate with small sample volumes of complex biological samples [1]. POC devices are used for prevention, control of disease outbreaks, and monitoring of fish health parameters in nonlaboratory environments such as aquaculture farms. Mass mortality in farmed fishes is due to the presence of bacterial infections; hence, rapid detection of such infections can achieve a faster diagnosis by point-of-care applications. Early detection of infections in fish has received extensive attention these days.

Streptococcosis, caused by *Streptococcus parauberis* (*S. parauberis*), is emerging as a major bacterial infection in olive flounder (*Paralichthys olivaceus*) fish [2]. The detection of *S. parauberis* mainly depends on the identification of clinical symptoms [3], conventional culture-based biochemical tests [4], and molecular tests such as polymerase chain reaction (PCR) based on the 23S rRNA region [2]. Although PCR is an established technique for the diagnosis of *S. parauberis*, the high sensitivity of the test may exceed clinical significance leading to false positives and targeting inappropriate specimen types, or suboptimal volumes of the specimen may result in false negatives [5].

Metabolites serve as physiological indicators of fish health status that help to understand host–pathogen interactions at small-molecule levels [6]. Metabolomics studies, such as liquid chromatography-tandem mass spectrometry (LC-MS) [7], optical spectroscopy [8,9], and nuclear magnetic resonance (^1^H-NMR) spectroscopy, have identified novel biomarkers for diagnosing bacterial infections in fishes. Although these techniques offer intrinsic advantages, such as high resolution and accuracy, the miniaturization of the equipment for point-of-care devices and their application remain a challenge [5]. Notably, electrochemical techniques are both highly selective and sensitive for target detection [10]. In particular, electrochemical biosensors are unique and more cost effective than other techniques described due to the fact of their minimal apparatus size, compactness, rapidness, and affordable instrumentation [11].

To construct an enzymatic Asp biosensor, a single enzyme (L-aspartase) or bi-enzyme (e.g., aspartate transaminase (AST) and glutamate oxidase (GluOx)) systems have been reported [12,13]. L-aspartase-based biosensors could convert the Asp to fumarate and ammonia gas. The enzymatically produced ammonia gas could be detected by the biosensor. This biosensor had limitations such as low specificity (easily affected by solvents and other gases) and longer response times [14]. Instead, as an alternative approach involving bi-enzymes, AST and GluOx are ideal for Asp quantification in a shorter amount of time. The enzymatic reaction involving AST catalyzes the conversion of Asp into oxaloacetate and glutamate in the presence of a coreactant, α-ketoglutarate (Equation (1)). From reaction 1, glutamate forms hydrogen peroxide (H_2_O_2_) as a byproduct through the enzymatic reaction catalyzed by GluOx. The formed H_2_O_2_ product can be easily detected by the nanozyme-modified sensing surface. Therefore, detecting H_2_O_2_ instead NH_3_ can be used for quantitative and qualitative Asp detection purposes. The enzymatic reaction cascade reaction is as follows:

(1) $$L-Asp+\alpha -ketoglutarate\;\overset{AST}{\to }\;Oxaloacetate+L-glutamate$$

(2) $$L-glutamate+O_{2}+H_{2}O\;\overset{GluOx}{\to }\;\alpha -ketoglutarate+NH_{3}+H_{2}O_{2}$$

(3) $$H_{2}O_{2}\to O_{2}+2H^{+}+2e^{-}$$

Thus, the produced H_2_O_2_ concentration is directly proportional to the added amount of Asp [8]. To reduce the working potential and to study the electroreduction process, Prussian blue (PB) nanozyme can be incorporated. PB nanozyme integration promotes signal amplification and sensitive H_2_O_2_ sensing, even at a lower applied potential. More importantly, the improvement in Asp detection with improved sensitivity at a low applied potential can be accomplished by PB integration [15]. Moreover, the extended π conjugation of graphene demonstrates high electrical conductivity, and enzymes immobilized on graphene retain their catalytic activity [16]. Specifically, reduced graphene oxide (rGO) is widely employed as a nano matrix because of its high loading capacity and simple synthesis procedure [17]. As the coimmobilization of two enzymes requires the recruitment of a nanosheet-like rGO matrix, polymer-dispersed rGO nano matrix inclusion is desirable.

Among various polymers, polystyrene-based copolymers possess improved properties, such as adhesivity, cohesivity, and high resistivity, in extreme temperature and pH conditions [18]. In addition, these copolymers serve as binders, suppressing enzyme leaching and providing easy reuse of the biocatalyst/enzyme for a few cycles. Therefore, rGO functionalization with polystyrene-based copolymers could be an inevitable approach, as it improves the stability of the biosensors [19].

**Scheme 1:**
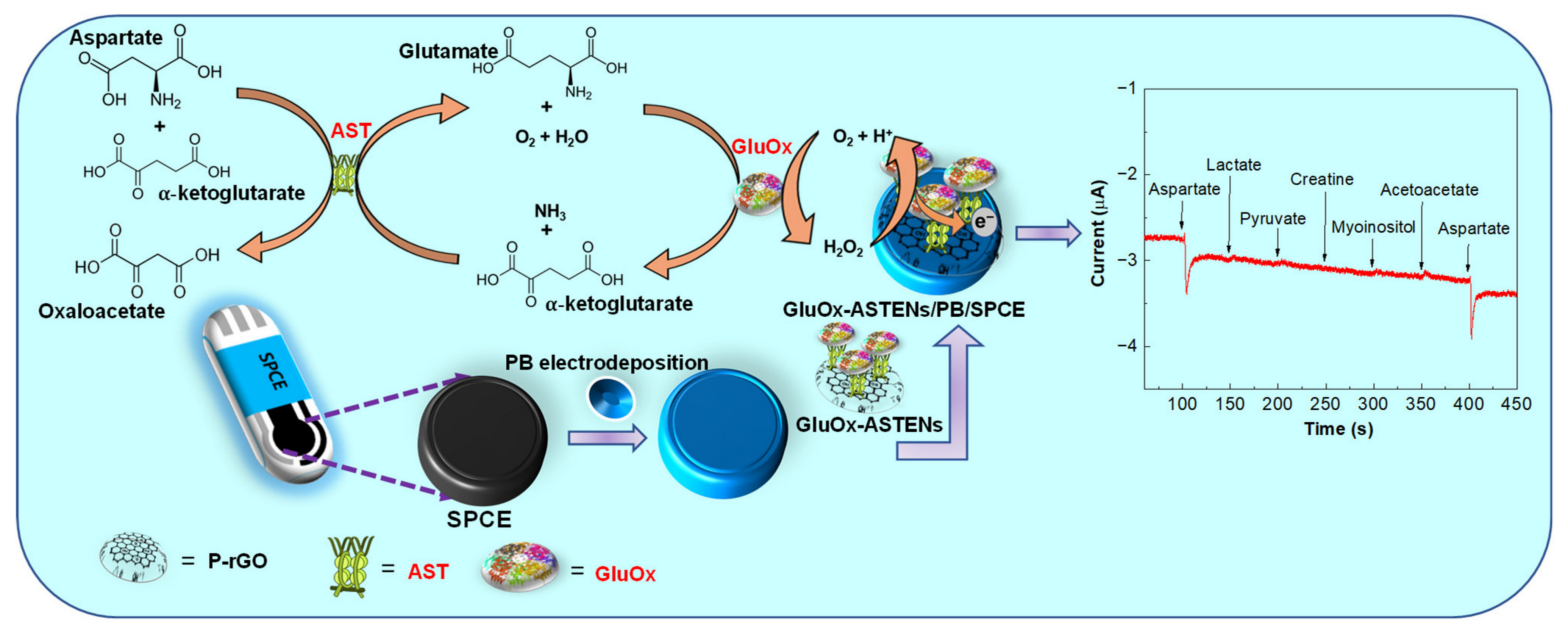
Schematic representation of the bi-enzyme-based Asp biosensor’s fabrication.

Initially, we aimed to identify the biomarkers in the spleen of olive flounder infected with *S. parauberis* using ^1^H-NMR. In ^1^H-NMR profiling of infected olive flounder fish, the significantly altered metabolites were glutarate, lactate, and aspartate (Asp). Recently our group reported a disposable amperometric lactate biosensor to detect lactate biomarker in fishes [19]. Furthermore, to target Asp, we designed an enzymatic biosensor for fish point-of-care applications. Firstly, the SPCE was electrodeposited with PB nanozyme (PB/SPCE). The rGO dispersed in a synthesized copolymer binder, (poly(sodium 4-styrene sulfonate-r-LAHEMA); (PSSL)), was termed P-rGO nanosheets. The P-rGO nanosheets combined with GluOx and AST enzymes were drop coated in two layers to favor electrostatic or hydrogen bonding interactions between the enzyme complexes and P-rGO nanosheets (GluOx–ASTENs). The P-rGO nanosheets not only provide a sufficient surface area for loading an appropriate number of bi-enzymes, but they also suppresses enzyme leakage, thus boosting its catalytic efficiency and enhancing the formation of a stable and selective sensing layer. The GluOx-ASTENs were co-immobilized over the nanozyme-modified biosensor and referred to as GluOx-ASTENs/PB/SPCE. A simple graphical representation of the designed aspartate biosensor is given as Scheme 1.

## 2. Materials and Methods

### 2.1. Reagents and Chemicals

This information is provided in the Supplementary Materials (S1.1.).

### 2.2. Instruments and Measurements

This information is provided in the Supplementary Materials (S1.2.).

### 2.3. Fabrication of Aspartate Biosensors

Initially, the synthesis of P-rGO and PB nanozyme modification onto the SPCE (PB/SPCE) was performed following a previous report [19]. To develop GluOx–ASTENs, equal volumes of P-rGO nanosheets (0.2 mg mL^−1^), GluOx, and AST were mixed and incubated for 7 min. As the enzymes are susceptible to inactivation, a uniform incubation time of 7 min was constantly followed. The P-rGO nanosheets were synthesized following a procedure reported elsewhere [19]. The P-rGO nanosheets were sufficient to entrap two the enzymes GluOx and AST simultaneously. A total of 6 µL of the developed GluOx–ASTENs was drop coated onto the PB/SPCE. The fabricated GluOx–ASTENs/PB/SPCEs were dried at 4 °C for 15 h before use. The PSSL polymer binder in P-rGO had adequate adhesive properties that enabled EN loading by physical adsorption.

## 3. Results and Discussion

### 3.1. Significant Metabolites in S. parauberis-Infected Fish

In the ^1^H-NMR analysis, a total of 21 metabolites were identified in the spleen of the olive flounder [19]. Principal component analysis (PCA) was performed to observe variations in the metabolic profiles, and the PCA score plot showed that the infected group was scattered and separated clearly from the control (Figure S1a). A partial least squares discriminant analysis (PLS-DA) was performed to demonstrate a clear separation between the control and the *S. parauberis*-infected group (Figure S1b). The validity of the PLS-DA model was confirmed as 0.838 for R^2^ and 0.746 for Q^2^. Using the VIP scoring system, the metabolites were ranked in which Asp was identified as the significantly increased metabolite (VIP > 1) (Figure S1c). Asp was further analyzed using a *t*-test, and a significant increase was noticed in the *S. parauberis*-infected group compared with the control (FDR < 0.05, Table 1). The concentration of the Asp biomarker in the control group was below 0.002 mM, whereas in the *S. parauberis*-infected group, the Asp concentration increased two-fold (Figure S2). To determine the diagnostic performance of Asp, the area under the curve (AUC) value of the receiver operating characteristic (ROC) curve was used, which showed good predictability with a high AUC above 0.80 (Table 1).

### 3.2. Physical Characterization

**Figure 1:**
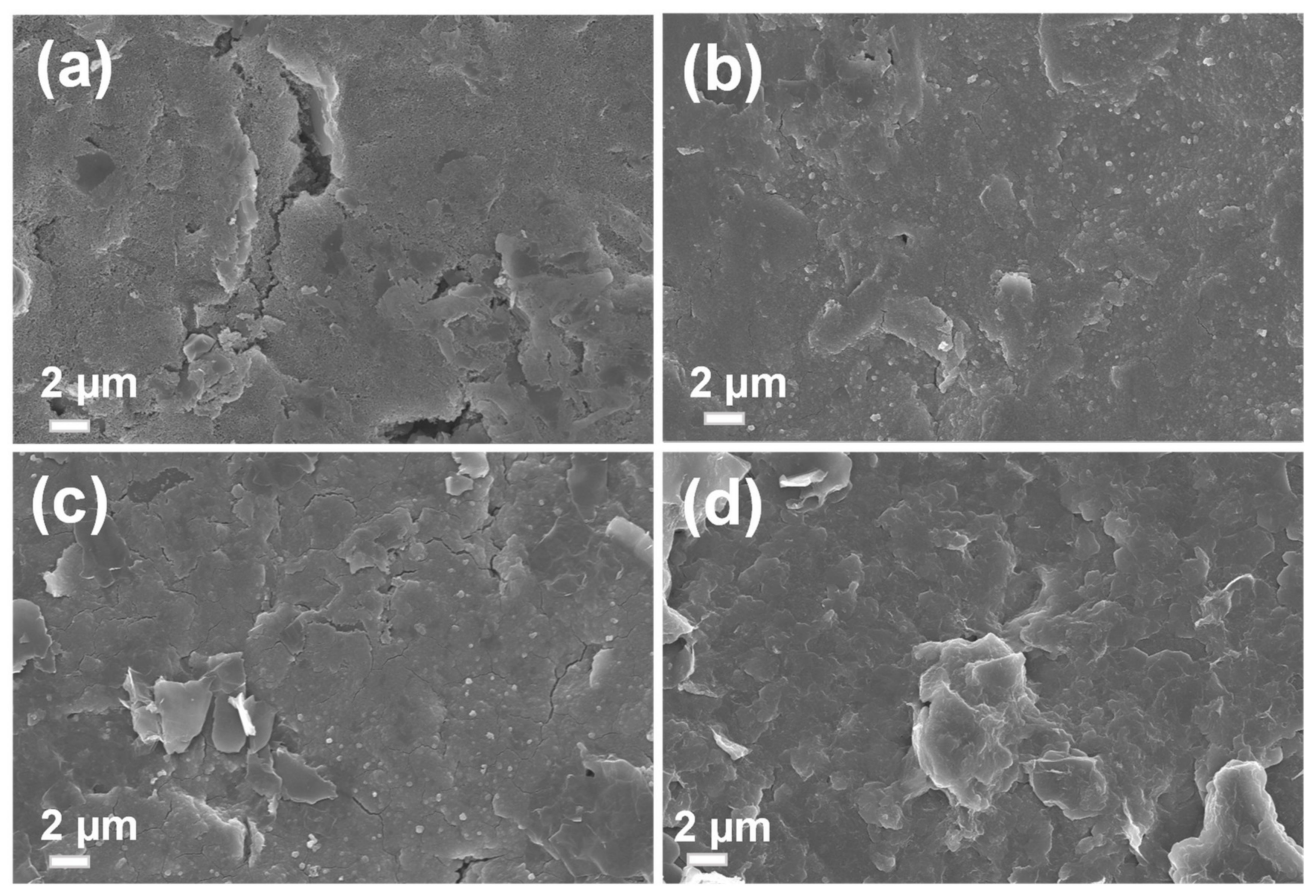
FE-SEM images of (**a**) bare SPCE; (**b**) PB/SPCE; (**c**) P-rGO/PB/SPCE; (**d**) GluOx-ASTENs/PB/SPCE; scale bar: 2 µm.

The morphology of the biosensors after stepwise modifications was characterized by field emission scanning electron microscopy (FE-SEM) as illustrated in Figure 1. The bare SPCE displayed a rough surface and an uneven morphology, whereas the surface of PB/SPCE was smoother with evenly distributed PB nanoparticles (Figure 1a,b). For P-rGO/PB/SPCE (Figure 1c), both PB nanoparticles and rGO nanosheet-like structures were observed. After GluOx–ASTENs immobilization, the surface became much smoother with aggregates (Figure 1d). These aggregates suggest good physical adsorption between the enzymes, AST-GluOx, and P-rGO nanosheets.

### 3.3. Electrochemical Characterization of the Differently Modified Biosensors

**Figure 2:**
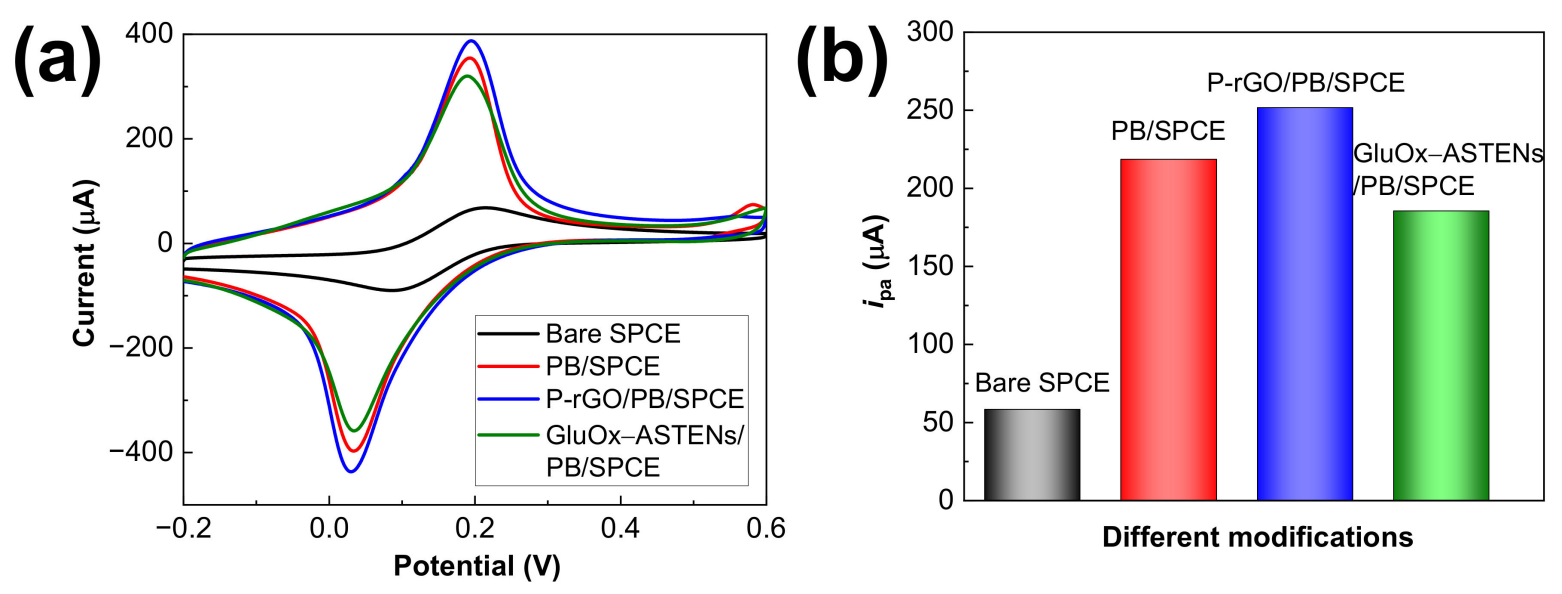
(**a**) Cyclic voltammograms of various biosensors in the presence of 5 mM K_3_[Fe(CN)_6_] in 0.1 M KCl at a scan rate of 50 mV s^−1^; (**b**) corresponding *i*_pa_ plot.

The electrochemical properties of various biosensors (i.e., bare SPCE, PB/SPCE, P-rGO/PB/SPCE, and GluOx-ASTENs/PB/SPCE) were evaluated by CV experiments. Figure 2a shows the cyclic voltammograms of the biosensors in 5 mM K_3_[Fe(CN)_6_] prepared in 0.1 M KCl at 50 mV s^−1^. All biosensors showed clear redox peaks. Except for bare SPCE, the other biosensors demonstrated much appreciable redox peaks due to the superior electron transfer characteristics of the PB, P-rGO, and GluOx-ASTENs modifications. Particularly, the P-rGO/PB/SPCE offered a higher redox peak current on the CV curve, indicating that this biosensor provided a high electrocatalytic activity toward the K_3_[Fe(CN)_6_] redox couple. Additionally, the anodic peak currents (*i*_pa_) of the biosensors were measured and plotted as shown in Figure 2b. The *i*_pa_ values for bare SPCE, PB/SPCE, P-rGO/PB/SPCE, and GluOx-ASTENs/PB/SPCE were 58.1, 218.5, 251.6, and 185.4 µA, respectively. In comparison with bare SPCE, PB/SPCE, P-rGO/PB/SPCE, and GluOx-ASTENs/PB/SPCE showed increased *i*_pa_ values, illustrating that the PB, P-rGO, and GluOx-ASTENs modifications improved the electroactive surface area of the biosensor. Especially, P-rGO/PB/SPCE demonstrated a maximum *i*_pa_ value. In comparison to P-rGO/PB/SPCE, the GluOx-ASTENs/PB/SPCE exhibited a mild decrease in its *i*_pa_ value, which was due to the immobilization of hydrophobic or negatively charged enzyme molecules that partially repelled the negatively charged K_3_[Fe(CN)_6_] redox probe toward the biosensors [20].

### 3.4. Enzyme Loading Amount

**Figure 3:**
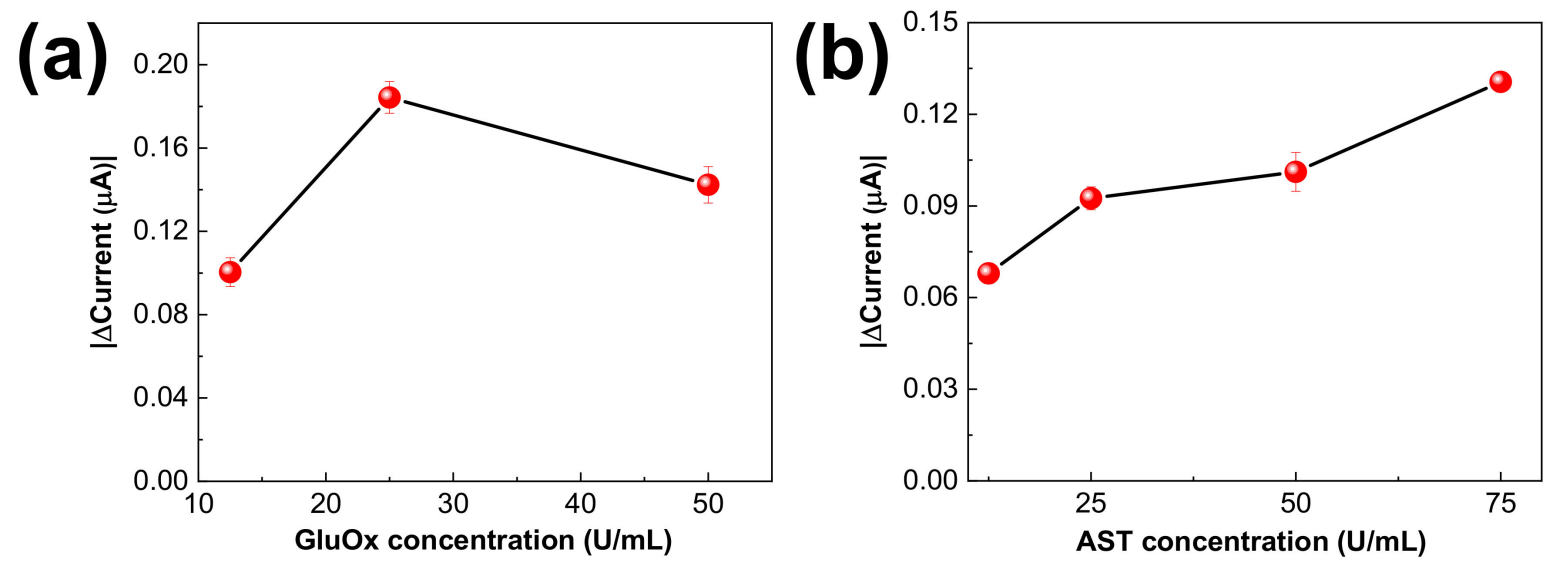
(**a**) Current changes to 0.1 mM glutamate using GluOxENs/PB/SPCE fabricated using various concentrations of GluOx. (**b**) Current changes against 0.8 mM Asp using GluOx–ASTENs/PB/SPCE fabricated using 25 U/mL GluOx and various concentrations (i.e., 12.5, 25.0, 50.0, and 75.0 U/mL) of AST in the presence of 150 µM ketoglutarate in the buffer.

The GluOx-ASTENs/PB/SPCE biosensors were optimized using both GluOx and AST enzyme loadings. Enzyme molecules apparently slow down the electron transfer, reducing the sensitivity of the biosensors. Therefore, optimizing the GluOx and AST loadings onto the P-rGO nanosheets using various concentrations of GluOx (i.e., 12.5, 25.0, and 50.0 U/mL) and AST (i.e., 12.5, 25.0, 50.0, and 75.0 U/mL) to prepare the GluOx-ASTENs were followed. The amperometric current responses increased as the GluOx concentration increased from 12.5 to 25.0 U/mL (Figure 3a). The higher GluOx loading concentration (i.e., 50.0 U/mL) demonstrated a slight decline in current responses. Therefore, 25.0 U/mL was chosen because of its improved current response or bioactivity. Likewise, as shown in Figure 3b, various loading concentrations of AST (i.e., 12.5, 25.0, 50.0, and 75.0 U/mL) were optimized in which 75.0 U/mL demonstrated increased bioactivity. Finally, 25.0 U/mL loadings of GluOx and ~75.0 U/mL AST loadings were chosen as the optimum compositions.

### 3.5. Optimization of α-Ketoglutarate

Apart from enzyme concentrations, the α-ketoglutarate (coreactant) concentration needed to be optimized to prevent insufficient concentration or excessive cofactor consumption issues in reaction 1 as mentioned in Section 1. Insufficient α-ketoglutarate is known to produce a very low glutamate concentration in reaction 1, while reaction 2 could be inhibited by higher concentrations of a-ketoglutarate [21].

**Figure 4:**
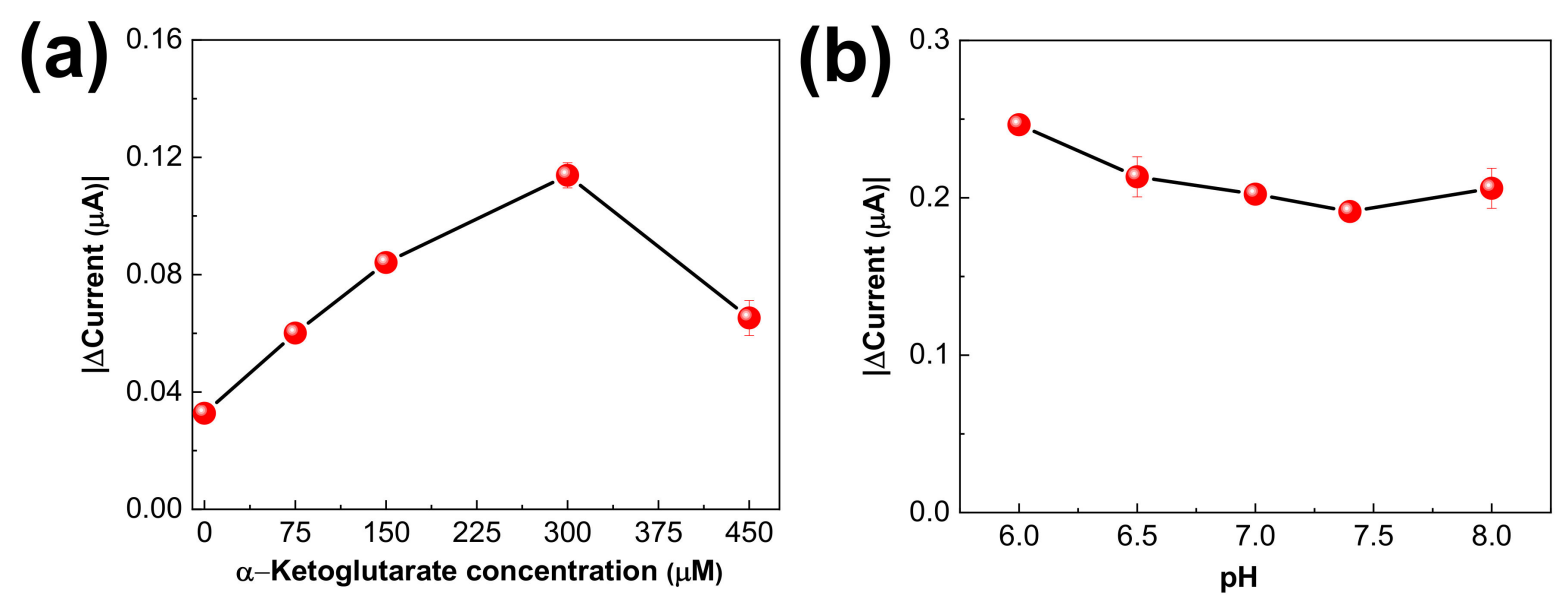
(**a**) Current changes against 0.8 mM Asp using GluOx–ASTENs/PB/SPCE fabricated using 25 U/mL GluOx and 62.5 U/mL AST in the presence of different concentrations of α-ketoglutarate (i.e., 0.0, 75.0, 150 and 300 µM) in the buffer. (**b**) Current changes across varied pH solutions on GluOx–ASTENs/PB/SPCE in 0.8 mM Asp in buffer at the applied potential of −0.1 V.

In CA, different concentrations of α-ketoglutarate (i.e., 75.0, 150, 300, and 450 µM) were tested against a fixed concentration of Asp (i.e., 800 µM) as represented in Figure 4a. The maximum AST current response was recorded for 300 µM of α-ketoglutarate. To prevent electrode fouling due to the presence of an excess concentration of α-ketoglutarate, 150 µM of α-ketoglutarate was selected as the optimal concentration.

### 3.6. Optimization of Buffer pH

The evaluation of the optimum pH value is a crucial factor for the improvement of the biosensor’s response, especially in a bi-enzyme/pH-dependent system to favor the formation of enzyme–substrate complexes. Hence, the effect of pH on GluOx-ASTENs/PB/SPCE was investigated by measuring the current response of the biosensors in 50 mM PBS between pH 6.0 and 8.0 after the addition of Asp. Maximum current responses were only observed near pH 6.0 as depicted in Figure 4b. The GluOx enzyme was catalytically active between pH 7.0 and 9.0, whereas the AST enzyme was active at pH 7.5, as per the manufacturer’s specifications. Both the enzymes are expected to show maximum activity at the basic pH range. Alternatively, the P-rGO nanosheets consisted of sulfonate and disulfide functionalities, which could possibly shift the pH to the acidic range. In addition, Asp has abundant COOH functional groups, which could slightly shift the pH to the acidic range. On the basis of these assumptions, for the pH optimization results, on account of enzyme stability and maximum bioactivity, pH 6.0 was taken as the most suitable pH for further experiments.

### 3.7. Asp Calibration Plot

**Figure 5:**
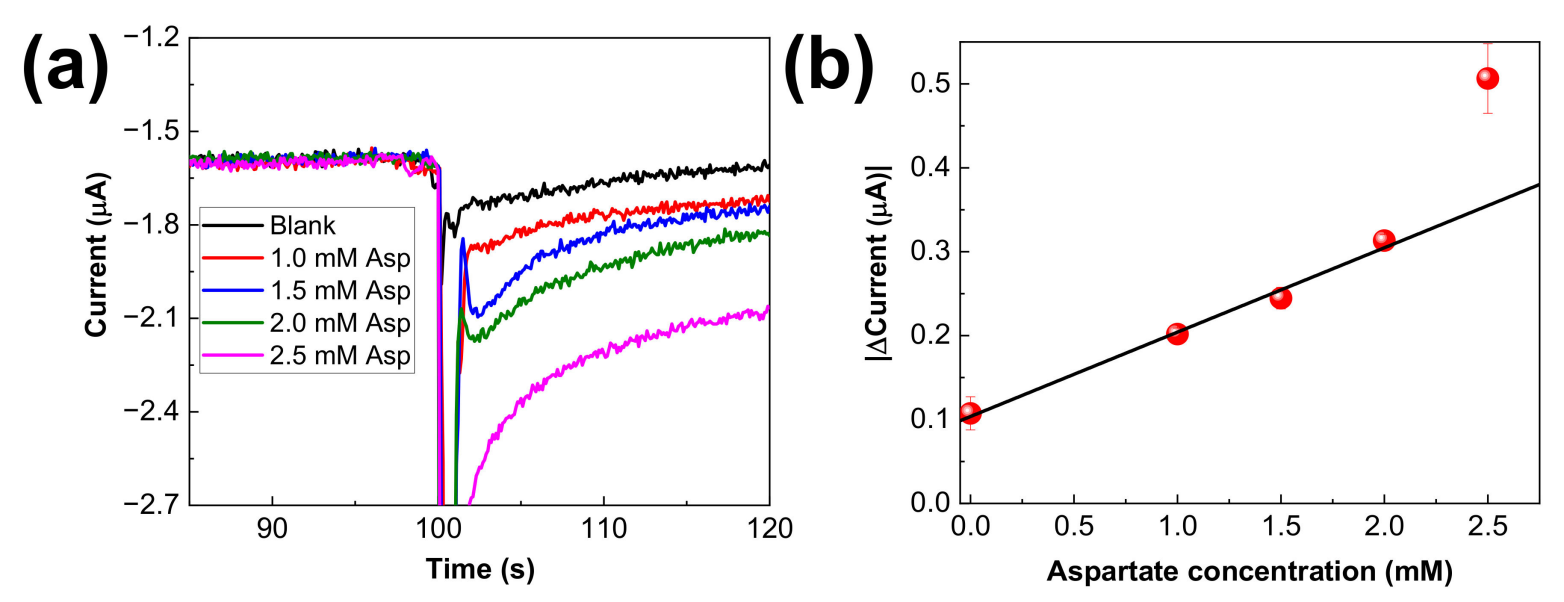
(**a**) Amperometric responses of GluOx–ASTENs/PB/SPCEs in 50 mM buffer (pH 6.0) with successive additions of different concentrations of aspartate at −0.1 V in the presence of 150 µM α-ketoglutarate. (**b**) Corresponding linear calibration curve.

CA measurements were performed for varied concentrations of Asp (i.e., 0.0, 1.0, 1.5, 2.0, and 2.5 mM) using GluOx-ASTENs/PB/SPCE under optimized conditions. As shown in Figure 5a, the current responses were stable and proportional to the added Asp concentration. The current responses after 20 s of Asp additions were measured and the calibration curve was plotted. (Figure 5b). The plotted results are the mean values of four measurements, with error bars signifying their standard deviation. The linear range was estimated to be 0–2.0 mM, and the regression equation was i (µA) = 0.1005 C_Asp_ (mM)–0.104 (R^2^ = 0.991) and a sensitivity of 0.8 µA mM^−1^ cm^−2^. The limit of detection (LOD) was 500 µM (S/N = 3). The characteristics of the biosensor were compared with the reported amperometric and potentiometric Asp biosensors summarized in Table 2. In comparison with other biosensors, the sensitivity of our biosensor was high. Yet for real sample applications, the LOD needs to be improved to a greater extent, and our group is contributing significant efforts toward improving the biosensor’s characteristics in detecting Asp.

Our future goal is to miniaturize the biosensor for real-time Asp measurements in fish samples. As the study requires an in-depth analysis of fish samples, efforts are being focused on developing a consistent and commercial point-of-care device to diagnose bacterial infections in fish samples.

### 3.8. Selectivity Study

**Figure 6:**
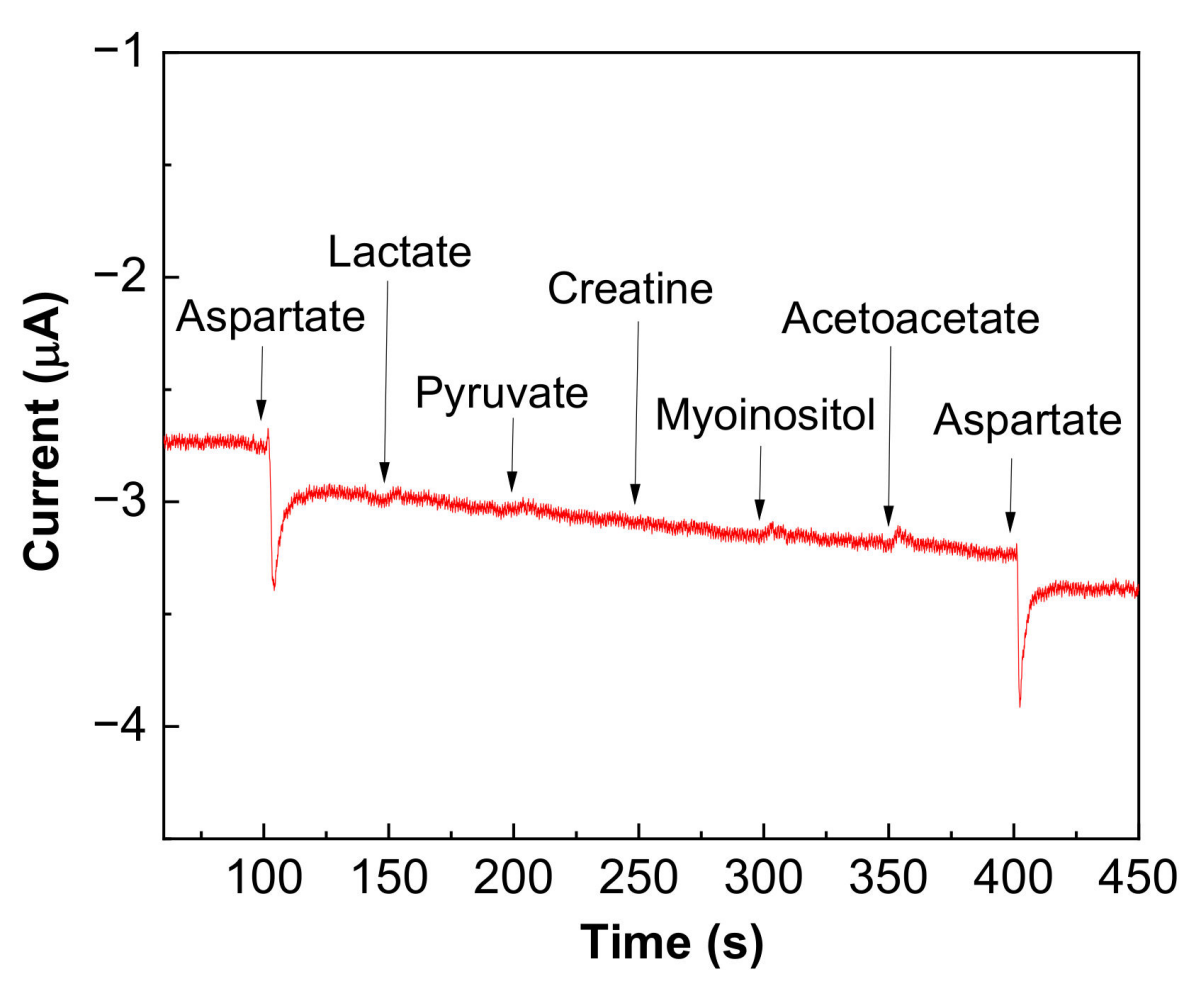
CA results of GluOx–ASTENs/PB/SPCE in 50 mM buffer (pH 6.0) at −0.1 V, with the additions of 0.8 mM Asp, lactate, pyruvate, creatine, myoinositol, and acetoacetate in the presence of 150 µM α-ketoglutarate.

A key parameter that should be considered for the practical application of the biosensor is the potential interfering effect of other substances present in the fish samples. To test the applicability of the proposed biosensor in the detection of Asp in the fish samples, the influence of common interferents present in the fish samples was studied. The investigation of the biosensor in the presence of 0.8 mM concentrations of common interferents (Figure 6) depicted a rapid current response to Asp additions (0.8 mM) that was not impacted by equal concentrations of coexisting interferents such as lactate, pyruvate, creatine, myoinositol, and acetoacetate. Notably, the anti-interference of the biosensor was probably due to the electronegative sulfonate and disulfide groups in the PSSL polymer that actively repelled negatively charged interferents such as lactate, pyruvate, and acetoacetate. These results suggest that selective Asp detection among various interferents is highly feasible using the proposed biosensor.

### 3.9. Reproducibility and Stability

Four GluOx–ASTENs/PB/SPCE (*n* = 4) were prepared under the same conditions to evaluate the reproducibility through CA experiments using 0.8 mM Asp. The relative standard deviation was 2.13% from four repeated measurements of the four biosensors as illustrated in Figure S3a. The stability of the biosensors was examined after storage at 4 °C for up to 2 weeks and tested weekly (*n* = 2). The loss of the initial current response was 24.7% and 36.2% after 1 and 2 weeks, respectively. Even after 2 weeks, the sensor retained nearly 63.8% of the current response as shown in Figure S3b. Therefore, the biosensor exhibited good reproducibility and stability in Asp detection.

## 4. Conclusions

Thus, the developed bi-enzyme Asp biosensor based on enzyme nanosheets was successfully applied to determine Asp concentration. The biosensor possessed a sensitivity of 0.8 μA mM^−1^ cm^−2^, a linear range of 1.0–2.0 mM, and an LOD of 500 μM for Asp detection. The utilized mild operating potential, −0.1 V vs. Ag/AgCl, not only improved the selectivity toward Asp but also suppressed the impact of susceptible interferents. In addition, the biosensor remained stable over two weeks of storage and demonstrated reasonable reproducibility. Therefore, the biosensor could be beneficial for monitoring Asp levels and could serve as a potential point-of-care device for diagnosing bacterial infection in fish farms. The ongoing experiments in fish samples may shed light on improving the design of point-of-care devices for bacterial infections in the future.

## Figures and Tables

**Table 1:** Significantly changed metabolites in the spleen of fish infected with *S. parauberis*.

**Metabolite**	**Concentration (mM, Mean ± SD)**		***p*** **-Value** **Adjusted by FDR**	**ROC Analysis**		
	Control	*S. parauberis*		AUC (95% CI)	Sensitivity	Specificity
Aspartate	0.019 ± 0.011	0.032 ± 0.008	0.036	0.844 (0.611–1)	100%	66.7%

**Table 2:** Comparison of the parameters obtained from various amperometric and potentiometric Asp biosensors.

Electrode	Enzyme Utilized	Applied Potential (vs. Ag/AgCl)	Electrochemical Technique	Target	Linear Range (mM)	LOD (µM)	Sensitivity (µA mM^−1^ cm^−2^)	Reference
AST-GluOx membrane/Pt	AST-GluOx	+650 mV	Amperometry	H_2_O_2_	0.001–0.2	-	-	[22]
AST-GluOx membrane/Pt	AST-GluOx	-	Amperometry	H_2_O_2_	0.005–0.2	-	-	[23]
Aspartase/Pt *	Aspartase	+0.3 V	Amperometry	H_2_O_2_	1–10	68	0.09	[12]
Aspartase/PAP *	Aspartase	-	Potentiometry	NH_3_	1.9–24	490	-	[13]
GluOx–ASTENs/PB/SPCE	AST-GluOx	−0.1 V	Amperometry	H_2_O_2_	1.0–2.0	500	0.8	This work

* Pt—platinum; PAP—polyazetidine prepolymer.

## Data Availability

Not applicable.

## Supplementary Materials

The following supporting information can be downloaded at: https://www.mdpi.com/article/10.3390/mi13091428/s1, Figure S1: (a,b) PLS-DA score plot and (c) VIP plot; Figure S2: The concentrations of identified Asp biomarkers in the control and the *S. parauberis* group through ^1^H-NMR; Figure S3: (a) Reproducibility of the GluOx-ASTENs/PB/SPCE and (b) stability of the GluOx-ASTENs/PB/SPCE after storage. Error bars = SD, *n* = 4.
