# Supplementary material for: Enzyme Nanosheet-Based Electrochemical Aspartate Biosensor for Fish Point-of-Care Applications
Source: Micromachines (Basel). 2022 Aug 29;13(9):1428. doi: 10.3390/mi13091428 (PMC9505169; doi:10.3390/mi13091428)
Supplement: Supplementary file 1 [file micromachines-13-01428-s001.zip › micromachines-1872484-supplementary.pdf]

Supplementary Material

Enzyme Nanosheet-Based Electrochemical Aspartate Biosensor for Fish Point-of-Care Applications

Thenmozhi Rajarathinam [1,†], Dinakaran Thirumalai [1,†], Sivaguru Jayaraman [1], Seonghye Kim [2], Minho Kwon [3], Hyun-jong Paik [3], Suhkmann Kim [2], Mijeong Kang [1] and Seung-Cheol Chang [1,*]

[1] Department of Cogno-Mechatronics Engineering, College of Nanoscience and Nanotechnology, Pusan National University, Busan 46241, Korea
[2] Department of Chemistry, Pusan National University, Busan 46241, Korea
[3] Department of Polymer Science and Engineering, Pusan National University, Busan 46241, Korea
* Correspondence: s.c.chang@pusan.ac.kr
† These authors contributed equally to this work.

S1.1. Reagents and chemicals

L-glutamate oxidase - 25 (GluOx, EC 1.4.3.11, 25 U/vial) from *Escherichia coli* species was procured from Cosmo bio, USA. Graphene oxide (GO) was obtained from Nanosolution (South Korea). The following reagents and chemicals were obtained from Sigma-Aldrich (USA): aspartate aminotransferase Type I, in ammonium sulfate suspension, (AST, EC 2.6.1.1, 200-500 units/vial) from porcine heart, L-aspartic acid, α-ketoglutarate, potassium ferricyanide ($K_3[Fe(CN)_6]$), 4-dimethyl aminopyridine (DMAP), iron (III) chloride ($FeCl_3$), sodium 4-styrene sulfonate (SS), 2-hydroxyethyl methacrylate (HEMA), sodium phosphate monobasic (NaH_2PO_4), hydrazine monohydrate, sodium phosphate dibasic (Na_2HPO_4), sodium chloride (NaCl), azobisisobutyronitrile (AIBN), N, N'-dicyclohexylcarbodiimide (DCC), lipoic acid (LA), dichloromethane (DCM), dimethyl sulfoxide (DMSO), magnesium sulfate ($MgSO_4$), and potassium chloride (KCl). Phosphate buffer (PBS) of 50 mM concentration and various pH was prepared using NaH_2PO_4, Na_2HPO_4, and KCl. All other reagents were of analytical grade and used without any further treatment. All solutions used in the experiments were prepared using triple distilled water.

S1.2. Instruments and measurements

Field emission scanning electron microscopy (FE-SEM) images were obtained using a Zeiss GeminiSEM 500 (Carl Zeiss Microscopy Deutschland GmbH, Oberkochen, Germany). Cyclic voltammetry (CV) experiments were done using an electrochemical workstation (CH Instruments Inc., Austin, USA, model 604E). chronoamperometry (CA) experiments were carried out using electrochemical equipment (Compactstat, Ivium Technologies B.V., Eindhoven, The Netherlands).

For CA, the biosensor was connected to a potentiostat, 60 μL of ketoglutarate prepared in PBS was placed onto the working electrode and potential of −0.10 V was applied. After a stable baseline response, 20 μL of Asp solution was added, and the obtained current responses were measured after 20 s. For the Asp calibration curve, the CA measurements were repeated using varied concentrations of Asp solutions.

S1.2.1 Experimental fish and S. parauberis challenge

Olive flounder were obtained from a commercial fish farm in South Korea and acclimatized to laboratory conditions for 2 weeks with seawater at 26 ºC. *Streptococcus parauberis* (*S. parauberis*) strain SpOF-3k serotype I was cultured on brain heart infusion agar (BHIA) containing 1% NaCl for 24 h. For *S. parauberis* infection, fish (*S. parauberis* infection group, n=20) were subcutaneously injected with *S. parauberis* strain at a final concentration of 4.3×10^3 colony-forming units/fish. The control group (n=20) was injected with the same volume of sterile saline. At 7 days post-challenge, fish were dissected after anesthesia with MS-222. The spleen was aseptically removed from each fish and frozen immediately using liquid nitrogen.

Infection was confirmed by homogenizing the isolated spleen, followed by serial dilution and culturing on BHIA for 24 h.

S1.2.2 ^1H NMR analysis

The individual spleen samples were lyophilized and homogenized for metabolites extraction. The homogenized samples were pooled to adjust the weight to an average of 60 mg for each control and *S.parauberis* infected group. Metabolite extraction was performed for each pooled sample using modified Bligh and Dyer's method was used[19]. The polar phase supernatant of each sample was collected and lyophilized. After lyophilization, the samples were redissolved with 700 μL of deuterium oxide (D_2O) containing 2 mM 3-(Trimethylsilyl) propionic-2,2,3,3-d4 acid sodium salt (TSP-d_4) and transferred into a 5 mm NMR tube. All samples were analyzed using a 600 MHz Agilent NMR spectrometer (Agilent, Santa Clara, CA, USA). A Carr-Purcell-Meiboom-Gill pulse (CPMG) sequence was used to suppress the peaks of water and macromolecule. Spectra were acquired with a 22.375 μs 90 pulse, 1 s relaxation delay, 3 s acquisition time, and 128 scans. The total acquisition time was 9 min 56 sec.

S1.2.3 Data Analysis

Identification and quantification of the metabolites were performed using Chenomx NMR suite 8.4 (Chenomx Inc., Edmonton, AB, Canada). The concentration of metabolites was calculated with reference to the 2 mM TSP peak. MetaboAnalyst 5.0, a web-based metabolomics analysis platform, was used for statistical analysis and biomarker analysis using the targeted profiling data. For multivariate statistical analysis, principal component analysis (PCA) and the partial least squares discriminant analysis (PLS-DA) were performed. Data were normalized by pareto-scaled. The PLS-DA model was evaluated with two parameters, R^2 (the goodness of fit) and Q^2 (the predictive ability). In PLS-DA model, the variable importance in projection (VIP) scores was used to identify significant metabolites which contributed to the group separation. The selected metabolites from PLS-DA model were analyzed using *t* test analysis adjusted to a false discovery rate (FDR) and the receiver operating characteristic (ROC) analysis.

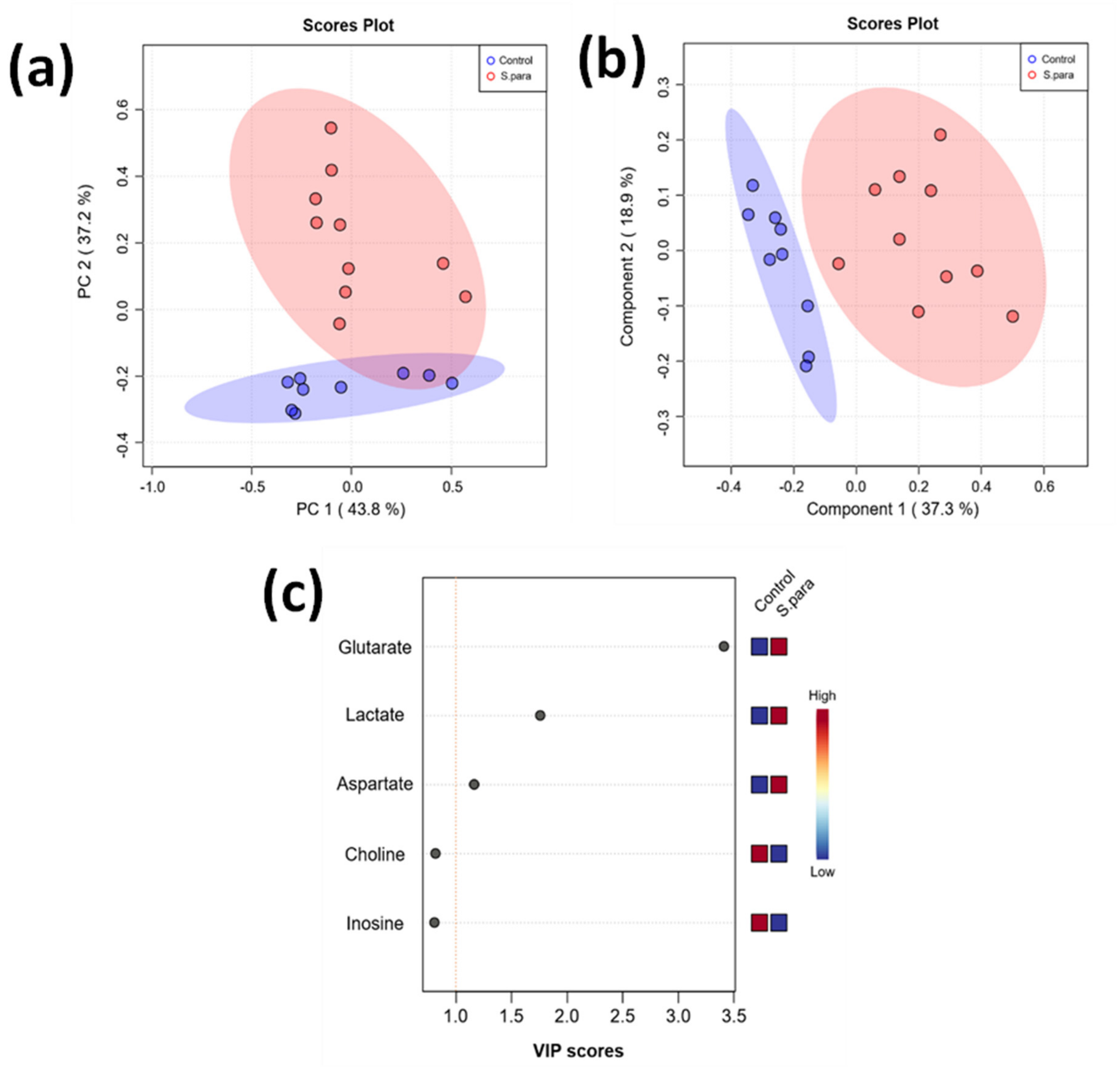

Figure S1. (**a & b**) PLS-DA score plot and (**c**) VIP plot. .

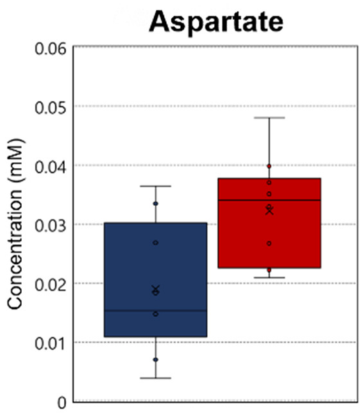

Figure S2. The concentrations of identified Asp biomarker in control and *S. parauberis* group through ^{1}H-NMR.

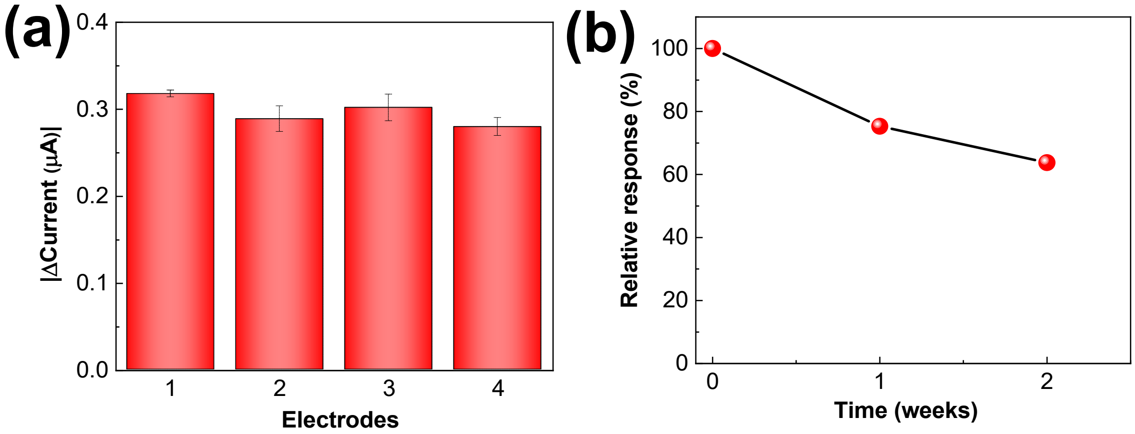

Figure S3. (**a**) Reproducibility of the GluOx-ASTENs/PB/SPCE and (**b**) Stability of the GluOx-ASTENs/PB/SPCE after storage. Error bars = SD, n = 4.
